# Theoretical adequacy, methodological quality and efficacy of online interventions targeting resilience: a systematic review and meta-analysis

**DOI:** 10.1093/eurpub/ckaa255

**Published:** 2021-07-07

**Authors:** Amanda Díaz-García, Marvin Franke, Rocio Herrero, David Daniel Ebert, Cristina Botella

**Affiliations:** 1 Department of Psychology and Sociology, Universidad de Zaragoza (Campus de Teruel), Teruel; 2 Clinical Psychology and Psychotherapy, Friedrich-Alexander University Erlangen Nuremberg, Erlangen, Germany; 3 CIBER Pathophysiology of Obesity and Nutrition (CB06/03), Carlos III Institute of Health, Madrid, Spain; 4 Department of Personality, Evaluation and Psychological Treatments, Universidad de Valencia, Valencia, Spain; 5 Universitat Jaume I, Castellón, Spain

## Abstract

**Background:**

There is a growing interest in the promotion of mental health, and concepts as resilience are re-emerging and taking relevance. In addition, Information and Communication Technologies can provide potential benefits in the field of mental health, and the treatment of mental disorders in particular. This study aims to synthesize the evidence of internet-based resilience interventions, analyzing the theoretical adequacy, methodological quality and efficacy.

**Methods:**

A systematic search was performed. The eligibility criteria stated for this article were: randomized controlled trials targeted at adults or adolescents and including any psychological intervention focussing on resilience in its rationale or design. Studies with direct (e.g. resilience scales) and proximal resilience measures (e.g. scales on well-being) were included. Risk of bias was assessed for each trial using Cochrane’s Collaboration Tool. Two reviewers worked independently in order to identify potential articles. A total of 11 articles were selected. A random-effects pooling model using the Hartung–Knapp–Sidik–Jonkman method based on direct and proximal resilience measures at post-test was used.

**Results:**

The overall effects of online resilience training compared to control groups at post-test were not significant; the effect size concerning the improvement of resilience was *g*=0.12 (95% CI: −0.14 to 0.38). In addition, a potential association between the type of outcome and the effect size could be revealed.

**Conclusions:**

The results of the present meta-analysis showed that the overall effect of online resilience trainings was not significant. Nonetheless, a tendency for a higher benefit for resilience was found in the studies with a clear assessment theory, indicating some promising effects.

**Registration Number:**

PROSPERO CRD42018083339.

## Introduction

In recent years, there has been growing interest in the promotion of core elements of mental health, such as well-being, positive functioning or quality of life. According to the World Health Organization’s definition, mental health is more than just the absence of mental illness.^1^ It also involves positive emotions, adaptive ways of interpreting reality, optimism and openness to the future.^2^ In this vein, the focus has been progressively shifting from deficit and psychopathology models to protective factors and strengths-based approaches, as well as psychological resilience.

Resilience has been conceptualized in different ways throughout the research literature. This concept has evolved from a trait-oriented approach (considered an intrinsic and stable attribute) determined by a certain personality type that helps individuals to cope with stress or adversity^3^ to an outcome-oriented approach that suggests that resilience is a behavioural outcome that can help people to recover when facing adversities.^4^ Finally, most recently, a process-oriented approach has increasingly been accepted, suggesting that resilience is a changeable and multidimensional, dynamic, and variable process of adaptation.^5^ However, there is an even greater array of possible ways to define resilience. For example, from the perspective of trauma, resilience is defined as efficacious adaptation, regardless of significant traumatic threats to personal and physical integrity.^6^ Luthar conceptualized resilience as a ‘dynamic process encompassing positive adaptation within the context of significant adversity’.^7^ Similarly, a concept analysis defined resilience as the process of effectively negotiating, adapting to, or managing significant sources of stress or trauma.^8^

Numerous definitions have been proposed in order to provide a conceptual framework that allows a better understanding of healthy development despite exposure to risk.^9^ Nevertheless, due to the heterogeneity in resilience definitions, no single accepted theoretical framework or universal operationalization of the resilience concept has been established.^10^ Even further, there are a wide number of studies without a clear reference to a resilience framework.^11^ Indeed, in an attempt to clarify the concept of resilience, some authors have recently reviewed and criticized the variety of definitions, concepts and theories of resilience.^12^

Despite the complexity of the concept, several interventions have been developed to enhance psychological resilience. Most of these interventions are based on cognitive-behavioural therapy, acceptance and commitment therapy, mindfulness-based therapy and problem-solving therapy. Besides, resilience-training programmes have been applied to clinical and non-clinical populations using different formats and settings.^13^ However, few interventions have a well-defined resilience model that can guide the mode of application of resilience interventions, and there is little consensus about the fundamental components for a programme to be considered a resilience intervention.^11^ In addition, further research is needed to test the efficacy and empirical evidence of these interventions.^14^

Recently, research has shown that Information and Communication Technologies (ICTs) are becoming more present in people’s lives and can also contribute to enhance happiness and well-being,^15^ which could among others be seen as proximal measures of resilience. ICTs may be used to promote resilience, increasing the possibilities of developing strategies to prevent mental disorders and reduce the negative effects of adversity on individuals’ mental health.^16^ As Riva^17^ pointed out, Positive Technology (Positive Psychology plus ICTs) can be used to increase wellness and generate strengths and resilience in individuals, organizations and society. Several advantages have been found in internet-delivered interventions related to accessibility, versatility, anonymity and scalability, which refers to expanding the effectiveness of interventions from the research setting to the real-world conditions.^18^ Besides, this delivery format facilitates the availability of evidence-based treatments.^19^ In this regard, the potential of resilience interventions may be enhanced by modifying the way that the intervention is delivered. It is not surprising, therefore, that some interventions focussed on enhancing psychological resilience have been developed online to reach a large number of people in need.^19^

To date, two systematic reviews^16^^,^^20^ and three meta-analyses^11^^,^^21^^,^^22^ have shown the efficacy of resilience interventions in adults showing positive effects of these programmes on different mental health outcomes and a positive impact on individual resilience. In addition, other systematic reviews have been conducted, targeting adolescents and young people, showing promising findings for using resilience-focussed interventions for reductions in depressive and anxiety symptoms and enhance resilience in this population.^23^^,^^24^ Furthermore, a narrative review was conducted on online positive interventions to foster resilience in the adolescent population, with the overall conclusions that more controlled studies are needed in this field.^25^ However, to the best of our knowledge, there are no reviews or meta-analyses focussing on resilience interventions applied over the internet.

Thus, the primary objective of this article is to synthesize the available evidence about the effectiveness of internet-based training interventions for improving resilience in adults or adolescents. In this regard, the efficacy of the interventions is analyzed in relation to their methodological quality and theoretical adequacy (e.g. showing a clear theoretical resilience framework and theoretically consistent study design).

## Methods

The Cochrane Collaboration guidelines^26^ were used as a guide to carry out this systematic review and meta-analysis. To assess the theoretical adequacy, several categories were selected to analyze the studies from a theoretical perspective. These categories were defined following previous studies addressing this topic.^14^ The study protocol was published in PROSPERO under registration number CRD42018083339.

### Eligibility criteria

Eligible studies were trials assessing the efficacy of interventions designed to develop or enhance resilience in clinical and non-clinical samples, for both adolescents and adults. We considered studies that reported measures of resilience at pre-post-treatment. We classified the measures as direct or proximal. A direct measure includes only resilience outcomes (e.g. Connor–Davidson scale or RS-14); while proximal measures are those that can be broadly associated with resilience, such as well-being, stress or quality of life^27^ (e.g. we used measures of well-being given that resilience is characterized by personal growth and shows an improvement of functioning).^8^ The decision to include non-resilience specific measures was made to reflect the current state of the literature and research, which is influenced by a broad understanding of the concept.

The studies had to be randomized controlled trials (RCT). For this article, we included studies published in English, Spanish or German to be eligible. Articles in other languages were excluded given the lack of expertise of the review team in these languages.

To be included in the meta-analysis, studies had to be targeted at adults or adolescents (11–18 years). Eligible studies included any psychological intervention focussing on resilience in its rationale or design. We included trials where the intervention was delivered through the internet or used blended treatment modalities (internet plus some face-to-face sessions). There were no restrictions based on the type of comparison condition used: waiting list, care-as-usual, placebo (e.g. a task where participant has to select a 5-petaled flower from a number of flowers), active (e.g. a serial addition test) and non-active control groups. The same applied to the setting, theoretical basis, content or length of the intervention, absence of follow-ups assessments or publication date.

### Search methods for identification of studies

A systematic search of the peer-reviewed literature was conducted using the following electronic databases: PubMed, PsycINFO, EBSCOhost and Cochrane Central Register of Controlled Trials. Additionally, Google Scholar, reference lists from relevant articles and previous meta-analysis were reviewed. A search regarding studies that are currently ongoing was performed by checking trial registries (ClinicalTrials.gov; isrctn.com). If the full-text or data of the so found articles were not available, the articles’ authors were asked to provide the needed information.

In order to perform the search, we used terms as resilience and associated constructs, like hardiness or cope, following the current resilience literature. This strategy was based on the search terms used in the previous systematic review of Leppin et al.^11^ Terms related to mobile-based trainings (e.g. smartphone, tablet) were also included as the delivery method used in these devices often is the internet, and the focus of this review was on the internet independent of the devices used.

The results of each search were combined with ‘and’, and the same search terms were used in the PubMed, PsycINFO EBSCOhost and Cochrane databases (see Supplementary material S1 for more detail on the searches used).

### Identification and selection of studies

In the first round of screening, two independent reviewers (A.D.-G. and R.H.) screened eligible articles by reading titles and abstracts in order to identify potentially relevant articles. Studies that were clearly ineligible were rejected. Inter-rater reliability was excellent (Kappa=0.76). In the second phase, reviewers independently assessed full-text versions of the relevant articles to determine final eligibility. Inter-rater reliability was excellent (Kappa =0.84). A total of eight criteria were established hierarchically, including whether the studies were RCTs, if their goal was to test an online application targeting resilience, the language and the comparison with a control condition, among others. Any disagreements were solved by consensus. In case in which consensus was not achieved, a third reviewer (C.B.) was consulted.

### Data extraction and management

Data regarding the included trials were extracted in a data extraction form. Information about the trials was collected, including trial’s authors, year of publication, study objective, population (patients, students, other), demographics (age, gender) of participants, setting, measures and information about the risk of bias and theoretical appropriateness. Information extracted: the number of participants approached, the number enrolled, randomization method, post-intervention means and standard deviations. The outcomes collected were self-reported measures of resilience, hardiness, quality of life or well-being.

One study compared two different resilience trainings to one control group.^28^ Therefore, according to the recommendation of the Cochrane Handbook for Systematic Reviews of Interventions,^26^ the two intervention groups were combined. Another study,^29^ however, reported two independent comparisons of two different interventions with their respective control groups. Since these studies are independent, they were treated as two separate comparisons.

When there was a direct resilience measurement used (e.g. CD-RISC), this outcome was extracted for the meta-analysis. Some studies, however, were targeted to improve resilience but only reported several proximal measurements as stress or well-being. If no direct measurement was present, it was decided by a consensus of the authors to include only the proximal outcome that was most appropriate for inclusion and closest to the defined understanding of resilience.

### Risk of bias within studies

Risk of bias was assessed for each trial independently by two team members (A.D.-G. and M.F.) using Cochrane’s Collaboration Tool.^26^ We considered the quality of the randomization sequence generation; whether treatment arm allocation was concealed; the blindness outcome; the degree and potential impact of missing data; the likelihood of incomplete reporting; and the presence of intent-to-treat analysis. Studies were defined as indicating either ‘low’, ‘high’ or ‘unclear’ risk of bias based on the aforementioned criteria. In case, where the intervention was explicitly intended to impact resilience and no measure of resilience was reported, we considered the study to be at high risk of selective reporting. Conflicts in judgement were resolved through discussion and consensus.

### Rating of the theoretical adequacy

An additional aim of the present meta-analysis was to not only test the effects of online intervention studies on the resilience construct but also to analyze the theoretical adequacy of each study. Therefore, following previous studies that focussed on these objectives, we developed an *ad hoc* classification framework to analyze each trial. We established three categories that were assessed using the same scale used for the risk of bias assessment (low risk, high risk and unclear risk): (i) presence of a background theory of resilience that sustains or guides the intervention; (ii) design of the intervention: how the theory is translated into the content of the programme; and (iii) construct assessment: how the intervention programme is assessed with respect to what is proposed in the theoretical model (see Supplementary table S2 for more details about criteria). These analyses were conducted by two reviewers independently (A.D.-G. and R.H.). One discrepancy arose between reviewers, and was resolved by consulting a third reviewer.

### Main analyses

Pooled analyses were performed using the ‘meta’^30^ and ‘metafor’^31^ packages in RStudio version 1.2.1335. Based on the expectation of true heterogeneity in effect sizes due to variations in the characteristics of the interventions, participants and outcome measures, a random-effects pooling model using the Hartung–Knapp–Sidik–Jonkman method^32^ was used. The main analysis was performed with all included studies, combining those with direct and proximal measures of resilience.

To allow the pooling of effects across different measures of similar constructs, between-group effect sizes (Hedges’*g*)^33^ were calculated from differences in post-intervention means and their respective standard deviations. The extracted means and standard deviations were recoded when higher scores of an outcome indicated worse results (e.g. proximal outcome: stress). This ensured that positive effect sizes always correspond to results that favour the intervention group. Due to a lack of consistency in follow-up examinations of resilience and the reduce number of participants that fulfilled the follow-up measures, no analyses could be performed with follow-up data.

For each meta-analysis, the Q-test was performed to examine dispersion. Moreover, heterogeneity was computed using the *I*^2^ statistic and its respective 95% confidence interval (95% CI) following the method described by Borenstein.^34^*I*^2^ values of 25%, 50% and 75% can tentatively be interpreted as a low, moderate and high heterogeneity, respectively.^35^ For better interpretability, the number needed to treat (NNT) was calculated for the derived Hedges’ *g* of the meta-analysis based on the formula of Kraemer and Kupfer,^36^ indicating the number of participants who needed to receive an intervention to have more success if the same number is exposed to the control condition.

Several sensitivity analyses were performed. Besides a meta-analysis using the widespread adopted DerSimonian–Laird estimator for *τ*2,^37^ one sensitivity analysis recalculated the results by including only those studies that directly measured resilience (omitting those with proximal resilience measures). Since there were no studies whose confidence interval did not overlap with the confidence interval of the pooled effect, outliers were controlled by removing studies whose point estimate of the effect size was outside the confidence interval of the pooled effect size. To examine the influence of individual studies on the overall effect, influence analyses (or ‘leave-one-out’ analyses) that omit one study at a time when calculating the pooled effect size^38^ were conducted.

Publication bias was examined using funnel plots, the Duval and Tweedi trim-and-fill procedure^39^ and Egger’s test.^40^

### Additional analyses

**Figure 1:**
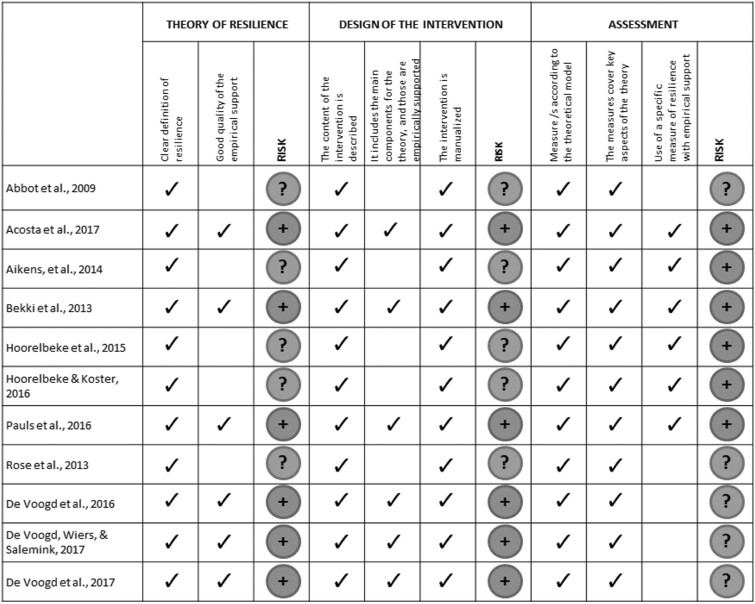
Theoretical appropriateness of studies. +, low risk of bias; –, high risk of bias; ?, unclear risk of bias

Since the total number of included comparisons exceeded *k*=10 (direct and proximal measures of resilience combined), subgroup analyses were performed for several study characteristics to assess the effect of potential moderators as well as identify sources of heterogeneity. Two subgroup analyses were conducted based directly on the extracted study characteristics (see Supplementary material S3): age of participants (adolescents vs. adults) and type of control group (active vs. non-active). Additionally, a third subgroup analysis depending on the risk of bias in included trials (rated based on the Cochrane RoB assessment tool) was performed (low risk vs. high risk). Lastly, three subgroup analyses were calculated based on the rating of the theoretical appropriateness regarding the theory of resilience, design of the intervention and assessment that is displayed in figure 1 (low risk vs. unclear risk) resulting in six subgroup analyses in total. These analyses used the mixed-effects model where the effect size for each subgroup is calculated based on a random-effects model, and the difference between the subgroups is tested with a fixed-effects model.

Two meta-regression analyses were conducted to determine the association between the effect size and the risk of bias rating (score) as well as the theoretical appropriateness rating (score). The latter reflects the sum of ‘low risk’ ratings throughout the three categories of theoretical appropriateness (see figure 1).

## Results

### Characteristics of included comparisons

The results of our search can be seen in the flowchart presented in Supplementary material S4. The three electronic databases searched generated a total of 1209 potential studies. Two additional trials were obtained through other sources. No ongoing studies were found by searching the trial registers. After retrieving duplicates, a total of 1010 studies were screened by title and abstract, resulting in 179 studies. Two independent researchers (R.H. and M.F.) reviewed full-text versions of these studies, providing the reasons for exclusion ranging from studies not available online (*n*=16), not being an RCT (*n*=68), full-text not available (*n*=1) through being other type of intervention (*n*=75) or targeting other population group (*n*=8). Thus, the final sample consisted of 11 RCTs selected for this meta-analysis. One of the studies^35^ reported two resilience trainings with their respective control groups; therefore it was analyzed separately, as described in the methods section, resulting in a total of *k* = 12 comparisons. Supplementary material S3 presents a summary of the characteristics of the trials included, as well as the theoretical basis for the interventions. In general, the purpose of the studies was to enhance resilience in diverse populations (e.g. veterans, employees and students). Interventions varied widely in design, duration and theoretical basis.

Most of the studies did not show a clear theoretical basis, drawing on broadly applicable strategies of coping, mindfulness, cognitive bias modification and/or cognitive-behavioural therapy. In addition, some of the studies included a follow-up assessment in the design (7 of 11 RCTs), 6 of the 11 RCTs included assessed resilience, and only 6 of the 11 RCTs reported a good quality of empirical support definition of resilience. A more detailed analysis of the theoretical appropriateness can be found in figure 1.

### Assessment of methodological quality

**Figure 2:**
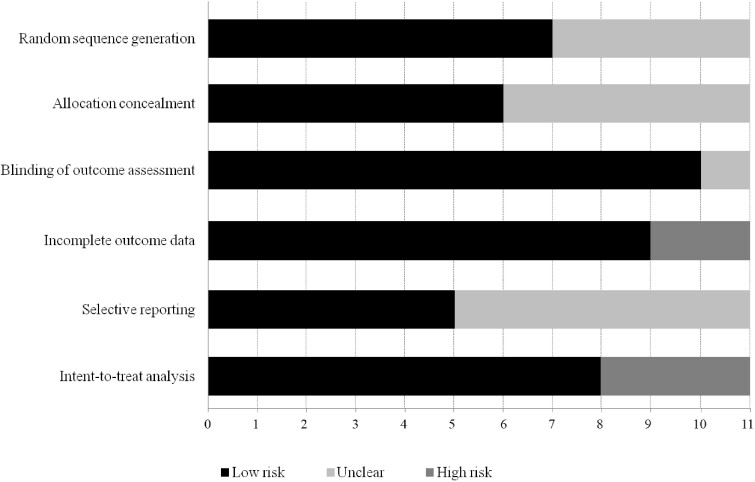
Methodological analysis of randomized controlled trials

Figure 2 summarizes the different aspects related to the methodological quality of the studies, following the Cochrane guidelines.^34^ Seven studies provided information about random sequence generation (e.g. computerized or permuted-block randomization), and six studies gave information about allocation concealment (e.g. ‘allocation was concealed’ or ‘allocation was secret to participants’). A total of 10 out of the 11 RCTs were judged at low risk in the blinding of the outcome assessment. Regarding incomplete outcome data, 2 of the 11 RCTs did not include an analysis of completers (i.e. participants considered dropout were not included in the final analyses). Finally, five studies provided data from all of the questionnaires administered at the beginning and end of the study, as well as information of data trial registration, and only three studies did not perform an intention-to-treat analysis.

### Meta-analyses

#### Effects of resilience training compared to the control group

**Figure 3:**
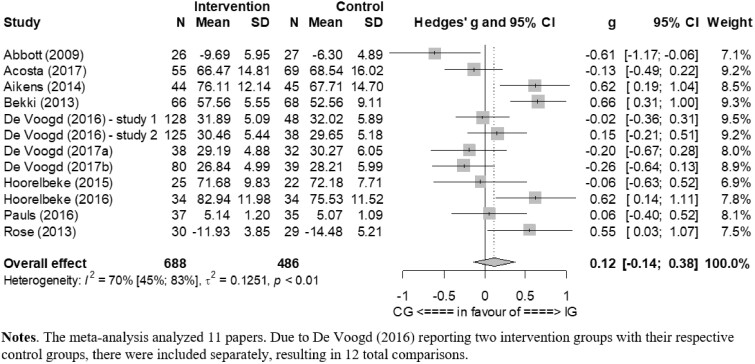
Forest plot for the effect sizes of the resilience interventions. Notes: the meta-analysis analyzed 11 papers. Due to De Voogd (2016) reporting two intervention groups with their respective control groups, there were included separately, resulting in 12 total comparisons

The overall pooled effect of the resilience training compared to the control groups at post-test was not significant (*P* = 0.32); the effect size concerning the improvement of resilience was small (*g* = 0.12; 95% CI: −0.14 to 0.38). This corresponds to an NNT of 14.67. Heterogeneity was moderate to high (*I*^2^=70%; 95% CI: 45–83). The effect sizes and 95% CI for each comparison are presented in figure 3. Since no study measured long-term effects, they could not be examined.

Several sensitivity analyses were performed. At first, the main analysis was repeated with the DerSimonian–Laird estimator for *τ*2 resulting in a similar effect of *g* = 0.12 with a more narrow 95% CI (*g* =−0.10 to 0.35). As another sensitivity analysis, the results were recalculated including only those studies that directly measured resilience (*k* = 6). The pooled effect of the intervention in those studies was still not significant (*P* = 0.11) but was related to a higher effect size of *g* = 0.30 (95% CI: −0.09 to 0.70). Lastly, excluding every study whose point estimate of the effect size was outside the confidence interval of the pooled effect size revealed a non-significant effect size of *g* = 0.00 (95% CI: −0.14 to 0.14) with no heterogeneity of *I*^2^=0 (95% CI: 0–36). More detailed information is displayed in table 1 and Supplementary materials S5 and S6.

**Table 1:** Effect of resilience training compared with control groups: Hedges’ *g*^a^

	***N*** ^c^	*g^a^* (95% CI)	*z* value	*I*² (95% CI)	***P*** ^b^	**NNT** ^d^
All studies	12^c^	0.12 (−0.14 to 0.38)	1.03 N.S.	69.78 (45.27–83.31)		14.67
Sensitivity analyses						
All studies DerSimonian–Laird estimator for *τ*²	12	0.12 (−0.10 to 0.35)	1.07 N.S.	69.78 (45.27–83.31)		14.60
Only direct measurement	6	0.30 (−0.09 to 0.70)	1.96 N.S.	68.90 (26.72–86.8)		5.89
Removed all outliers (point estimate outside pooled CI)	5	0.00 (−0.14 to 0.14)	0.02 N.S.	0 (0–36.19)		–^e^
Subgroup analyses						
Participants					0.119	
Adolescents	4	−0.07 (−0.36 to 0.22)	−0.72 N.S.	0 (0–82.57)		–^e^
Adults	8	0.23 (−0.16 to 0.61)	1.38 N.S.	74.32 (48.03–87.32)		7.85
Control group					0.872	
Active	7	0.13 (−0.16 to 0.42)	1.09 N.S.	47.89 (0–77.99)		13.68
Non-active	5	0.09 (−0.59 to 0.77)	0.35 N.S.	83.75 (63.3–92.81)		20.64
Risk of bias					0.395	
High risk	2	0.01 (−0.69 to 0.72)	0.24 N.S.	0^f^		133.85
Low risk	10	0.14 (−0.18 to 0.46)	1.01 N.S.	74.90 (53.19–86.55)		12.6
Theory of resilience					0.473	
Low risk	7	0.05 (−0.24 to 0.34)	0.39 N.S.	63.93 (18.48–84.04)		38.48
Unclear risk	5	0.24 (−0.44 to 0.92)	0.99 N.S.	76.08 (41.55–90.21)		7.4
Design of intervention					0.489	
Low risk	6	0.04 (−0.31 to 0.4)	0.31 N.S.	69.94 (29.58–87.17)		41.26
Unclear risk	6	0.21 (−0.30 to 0.73)	1.06 N.S.	71.53 (33.96–87.73)		8.42
Theory of assessment					0.095	
Low risk	6	0.30 (−0.09 to 0.70)	1.96 N.S.	68.90 (26.72–86.8)		5.89
Unclear risk	6	−0.06 (−0.45 to 0.33)	−0.39 N.S.	57.55 (0–82.84)		–^e^

Notes: CI, confidence interval; NNT, numbers needed to treat; N.S., not significant (*P*>0.05).

a According to the random-effects model.

b The *P*-values indicate whether the difference between the effect sizes in the subgroups is significant.

c The meta-analysis analyzed 11 articles but since 1 article reported 2 studies, there were included separately.

d The 95% CI for NNT was not calculated because the lower limit was always below zero.

e The NNT was not calculated since it was below zero.

f Confidence interval of *I*^2^ could not be calculated due to the small number of comparisons.

g No analysis was performed since the number of comparisons was below 3.

The results of the leave-one-out-analysis showed low effect sizes from *g* = 0.07 to *g* = 0.18 and heterogeneity values from *I*^2^=62% to *I*^2^=73% (for details see Supplementary material S7). Inspecting the funnel plot and Duval and Tweedie’s trim-and-fill procedure indicated that there was no publication bias (see Supplementary material S8). Moreover, Egger’s test did not show any indication of an asymmetric funnel plot (intercept: −0.80; 95% CI: −6.84 to 5.23; d*f* *=* 10, *P* = 0.80).

#### Subgroup and meta-regression analyses

A series of subgroup analyses were conducted to examine associations between the effect sizes and the characteristics of the study. There was no indication that the effect size was significantly associated with the age of participants (adolescents vs. adults), type of control group (active vs. non-active) or the risk of bias rating (low risk vs. high risk) since there were no statistically significant differences between the subgroups (*P* = 0.12, *P* = 0.87 and *P* = 0.40, respectively). The subgroup analyses regarding the theory of resilience (low risk vs. unclear risk) and the design of the intervention (low risk vs. unclear risk) yielded similar results and showed no statistically significant differences (*P* = 0.47 and *P* = 0.49) between the subgroups (for details see Supplementary materials S9–S13 and table 1).

Looking at the theory of assessment, however, a potential association between the theoretical appropriateness of the assessment and the effect size could be revealed (*P* = 0.095). Six studies were rated ‘low risk’, whereas five studies (six comparisons) were rated ‘unclear risk’ regarding the assessment (categorization see figure 2). Looking at the effects on the subgroup level, a statistical trend (*P* = 0.11) regarding the small effect of *g* = 0.30 (95% CI: −0.01 to 0.62) was found in the group of studies rated ‘low risk’. Studies in this subgroup showed a moderate to high heterogeneity of *I*^2^=68.90% (95% CI: 26.72–87.17). The overall effect in the group of studies ‘unclear risk’ rating regarding the assessment was not significant (*g*=−0.06; 95% CI: −0.45 to 0.33). Studies in this subgroup showed moderate heterogeneity of *I*^2^=57.55% (95% CI: 0–82.84). The corresponding forest plot is displayed in Supplementary material S14. The results of all subgroup analyses are summarized in table 1.

These findings are reflected in the associated meta-regression analyses with Hedges’ *g* as the dependent variable. The analysis did not indicate associations between characteristics of the studies and effect sizes *e* (slope=−0.04, 95% CI: −0.15 to 0.07, *P* = 0.45) or between the theoretical appropriateness assessment (score) and the effect size (slope=−0.002, 95% CI: −0.29 to 0.29, *P* = 0.99).

## Discussion

The results of the present meta-analysis showed that the overall effect of online resilience training on direct and proximal resilience measures was not significant. The results of the meta-analysis need to be interpreted with caution, however. The main result combined studies that directly measured resilience and those with proximal resilience measures. Even though this is reflective of the current state of research, it needs to be noted, that this limits generalizability. The sensitivity analysis including only studies directly measuring resilience indicates, however, that studies with a clear theoretical background and measurement show certain, but non-significant effect. This underlines the need of more resilience interventions with a stronger theoretical foundation, concerning not only in the content of the intervention itself but also in the way its effects on resilience are measured.

Several reasons can be identified to explain these findings, such as the high heterogeneity among the studies or the lack of an appropriate theory supporting the resilience programme. Specifically, concerning the theoretical basis, several studies showed the lack of a clear concept of resilience throughout the study (see Supplementary table S2). Whereas some studies made an effort to clarify the basis for the study question and intervention, others only mentioned the word ‘resilience’ and instead described a more general mental health promotion intervention. Future studies examining resilience should be aware of the theoretical literature on this topic and try to establish a clear theoretical basis, in order to achieve more conclusive results about the effectiveness of theoretically based resilience interventions.

In this review, the synthesis of the literature on online resilience interventions revealed the widespread use of questionnaire-based assessments of resilience. Although this might be a viable option, especially in the area of online-based interventions, it should be noted that it is difficult to measure resilience in this way. The current meta-analysis showed that there is a tendency for studies with a low risk of bias due to the theory of assessment to have better overall effects than studies with an unclear theory of assessment, possibly because of a close match between what the intervention was supposed to improve and what the study actually measured. Moreover, using a direct resilience measure could be an indicator of more theoretically based development of the interventions and studies themselves, with a clear goal stated from the beginning. However, the results of the other subgroup analysis did not show any indication that the theoretical background determined the effectiveness of the intervention. Future studies are needed to further determine what kind of intervention helps to improve resilience. Additionally, because the concept of resilience addresses the ability to cope with stressful situations, there should be a higher proportion of studies examining resilience in the long- term, directly assessing the way stressful situations were handled as opposed to the use of questionnaires focussing on general statements (e.g. CD-RISC, RS-14). Beside, another plausible strategy in order to complement the measurement of resilience could be achieved with an experimental stress paradigm (e.g. Trier Social Stress Test, Montreal Imaging Stress Task). Future longitudinal studies are needed to expand the current literature and do justice to the long-term process of changes in resilience.

This review has some limitations. The first is related to the low number of studies obtained, which could be due to the novelty of the field of resilience programme delivery through an online format. This could be an obstacle to drawing conclusions with a high level of reliability. Second, given that the review has been conducted with three databases, and there were language restrictions (English, Spanish and German), some studies might have been left out. Finally, another limitation is related to the outcome measures and the heterogeneity of the resilience concept across the different studies, which can have an impact on the generalization of the current results.

In conclusion, as far as we know, although some systematic reviews of resilience have been carried out, none of them has been focussed on the use of the internet to deliver resilience programmes. The results obtained emphasize the need to continue to clarify the concept of resilience, design studies with better methodologies and conduct more research on the effectiveness of these intervention programmes in promoting resilience.

To synthesize the available evidence about the effectiveness of internet-based training interventions for improving resilience carries important implications for mental health policy and practice. First, these interventions are expected to deal with challenges for the health-care system, focussing on the prevention of disorders and maintenance of good health instead of solely treatment of deficits. Consequently, the knowledge of the existing evidence of the efficacy of these interventions appears to be crucial to optimize the design of future studies in the field. Second, the development of these online interventions presents an opportunity to public health agencies to disseminate evidence-based psychological treatment among the population, and thus offer a more cost-effective option to reach people on a broader scale. Participation by health-care providers in these efforts is of utmost importance. Finally, findings from this meta-analysis also add information about the enormous need to clarify the concept of resilience to ensure that patients that are seeking to enhance their resilience receive a high-quality intervention, tested and validated for that purpose.

## Note

During the review process, there were differences between the protocol registration and this article. These differences are summarized in Supplementary material S15.

## Supplementary data

Supplementary data are available at *EURPUB* online.

## Supplementary Material

ckaa255_Supplementary_Data
