# Supplementary material for: Theoretical adequacy, methodological quality and efficacy of online interventions targeting resilience: a systematic review and meta-analysis
Source: Eur J Public Health. 2021 Jul 7;31(Suppl 1):i11–8. doi: 10.1093/eurpub/ckaa255 (PMC8266533; doi:10.1093/eurpub/ckaa255)
Supplement: ckaa255_Supplementary_Data [file ckaa255_supplementary_data.zip › ckaa255-suppl_data/S15_Additional information.docx]

**S15. Additional information.**

*Differences between the final review and the protocol*

The study registration stated that, if there are enough studies included in the meta-analysis, subgroup analysis will be conducted on:

- Group of participants (e.g., patients populations versus general population versus students versus employees),
- Type of comparator (e.g., active versus non-active control groups),
- Setting of the intervention,
- Theoretical foundation of resilience intervention (e.g., cognitive behavioural therapy versus mindfulness-based therapy versus problem-solving training),
- The quality of study/risk of bias.

Due to the information provided in the included studies as well as the way the studies were conducted, it was necessary to differ from the subgroup analyses stated in the protocol:

- Since only 11 studies were included, the group of participants was changed to the analysis of studies performed with adolescents and studies performed with adults.
- Active versus non-active control groups could be performed as planned.
- Setting of the intervention could not be analysed as planned, due to the limited information provided by the studies
- The theoretical foundation of the resilience intervention was adapted in accordance with the new theoretical appropriateness assessment regarding the design of the intervention
- The quality of the studies/ risk of bias could be performed as planned.
