# Supplementary material for: Theoretical adequacy, methodological quality and efficacy of online interventions targeting resilience: a systematic review and meta-analysis
Source: Eur J Public Health. 2021 Jul 7;31(Suppl 1):i11–8. doi: 10.1093/eurpub/ckaa255 (PMC8266533; doi:10.1093/eurpub/ckaa255)
Supplement: ckaa255_Supplementary_Data [file ckaa255_supplementary_data.zip › ckaa255-suppl_data/S16_References of studies included.docx]

**S16_References of studies included in the meta-analysis**

1. Abbott J-A, Klein B, Hamilton C, Rosenthal A. The impact of online resilience training for sales managers on wellbeing and performance. E-Journal Appl Psychol. 2009;5(1):89–95.
2. Acosta, MC, Possemato, K, Maisto, SA, Marsch, LA, Barrie, K, Lantinga, L, ... & Rosenblum, A. Web-delivered CBT reduces heavy drinking in OEF-OIF veterans in primary care with symptomatic substance use and PTSD. Behavior therapy. 2017; 48(2), 262-276.
3. Aikens, KA, Astin, J, Pelletier, KR, Levanovich, K, Baase, CM, Park, YY, Bodnar, C. M. Mindfulness goes to work: Impact of an online workplace intervention. Journal of Occupational and Environmental Medicine. 2014;56(7), 721-731.
4. Bekki JM, Smith ML, Bernstein BL, Harrison C. Effects of an Online Personal Resilience Training Program for Women in Stem Doctoral Programs. J Women Minor Sci Eng. 2013;19(1):17–35.
5. De Voogd EL, Wiers RW, Prins PJM, De Jong PJ, Boendermaker WJ, Zwitser RJ, Salemink E. Online attentional bias modification training targeting anxiety and depression in unselected adolescents: short-and long-term effects of a randomized controlled trial. Behaviour Rresearch and Ttherapy. 2016; 87, 11-22.
6. De Voogd, EL, Wiers, RW, Salemink, E. Online visual search attentional bias modification for adolescents with heightened anxiety and depressive symptoms: A randomized controlled trial. Behaviour research and therapy. 2017;92, 57-67.
7. De Voogd EL, de Hullu E, Burnett Heyes S, Blackwell SE, Wiers RW, Salemink E. Imagine the bright side of life: A randomized controlled trial of two types of interpretation bias modification procedure targeting adolescent anxiety and depression. PLoS One. 2017; 12(7): e0181147.
8. Hoorelbeke, K, Koster, EH, Vanderhasselt, MA, Callewaert, S, Demeyer, I. The influence of cognitive control training on stress reactivity and rumination in response to a lab stressor and naturalistic stress. Behaviour research and therapy. 2015;69, 1-10.
9. Hoorelbeke, K, Koster, EH. Internet-delivered cognitive control training as a preventive intervention for remitted depressed patients: Evidence from a double-blind randomized controlled trial study. Journal of consulting and clinical psychology. 2017;85(2), 135.
10. Pauls, N, Schlett, C, Soucek, R, Ziegler, M, Frank, N. Resilienz durch Training personaler Ressourcen stärken: Evaluation einer web-basierten Achtsamkeitsintervention. Gruppe. Interaktion. Organisation. Zeitschrift für Angewandte Organisationspsychologie (GIO). 2016;47(2), 105-117.
11. Rose, RD, Buckey Jr, JC, Zbozinek, TD, Motivala, SJ, Glenn, DE, Cartreine, JA, Craske, MG. A randomized controlled trial of a self-guided, multimedia, stress management and resilience training program. Behaviour Research and Therapy. 2013; 51(2), 106-112.
