# Supplementary material for: Theoretical adequacy, methodological quality and efficacy of online interventions targeting resilience: a systematic review and meta-analysis
Source: Eur J Public Health. 2021 Jul 7;31(Suppl 1):i11–8. doi: 10.1093/eurpub/ckaa255 (PMC8266533; doi:10.1093/eurpub/ckaa255)
Supplement: ckaa255_Supplementary_Data [file ckaa255_supplementary_data.zip › ckaa255-suppl_data/S1_Search strategy for the electronic databases.docx]

S1. **Search strategy for the electronic databases**

**PubMed:**

(“Resilien*”[All Fields] OR “hardiness”[All Fields] OR “cope”[All Fields] OR “coping”[All Fields] OR “psychological resilience”[All Fields]) AND (“psychological intervention”[All Fields] OR "psychological interventions"[All Fields] OR “building”[All fields] OR “enhance*”[All fields] OR “train*”[All fields] OR “educat*”[All fields] OR “teach*”[All fields] OR “increas*”[All fields] OR “promot*”[All fields] OR “prevent*”[All fields] OR “intervention” OR “interventions”) AND ("Internet based"[All fields] OR "Online"[All fields] OR "Online Therapy"[All fields] OR "Internet"[All fields] OR "Internet"[All fields] OR "Internet treatment"[All fields] OR "Web based"[All fields] OR "Internet intervention"[All fields] OR "Internet delivered"[All fields] OR "iCBT"[All fields] OR "website"[All fields] OR "computer"[All fields] OR "computer based"[All fields] OR "computer delivered"[All fields] OR "computer administered"[All fields] OR "technology"[All fields] OR "technology assisted"[All fields] OR "ICT"[All fields] OR "computerized"[All fields] OR "computerised"[All fields] OR "TELEhealth"[All fields] OR "eHealth"[All fields] OR "mobile"[All fields] OR "smartphone"[All fields] OR "tablet"[All fields])

**PsycINFO EBSCOhost:**

Any Field: resilien$ OR MeSH: psychological resilience OR Any Field: resilience intervention OR Any Field: resilience interventions OR Any Field: resilience-building program OR Any Field: resilience-based intervention OR Any Field: resilience-train$ OR Any Field: positive psychology OR Any Field: positive psychology intervention OR Any Field: positive psychology interventions OR Any Field: positive intervention OR Any Field: positive interventions OR Any Field: positive psychological intervention OR Any Field: positive psychological interventions OR Any Field: positive psychology program OR Any Field: positive component OR Any Field: positive components OR Any Field: positive psychology programs AND Any Field: Internet based OR Any Field: Online OR Any Field: Online Therapy OR Any Field: Internet OR MeSH: Internet OR Any Field: Internet treatment OR Any Field: Web based OR Any Field: Internet intervention OR Any Field: Internet delivered OR Any Field: iCBT OR Any Field: website OR Any Field: computer OR Any Field: computer based OR Any Field: computer delivered OR Any Field: computer administered OR Any Field: technology OR Any Field: technology assisted OR Any Field: ICT OR Any Field: computerized OR Any Field: computerised OR Any Field: TELEhealth OR Any Field: eHealth OR Any Field: mobile OR Any Field: smartphone OR Any Field: tablet

**Cochrane Central Register of Controlled Trials (CENTRAL):**

(“Resilien*”[All Fields] OR “hardiness”[All Fields] OR “cope”[All Fields] OR “coping”[All Fields] OR “resilience, psychological”[MeSH Terms]) AND (“resilience intervention”[All Fields] OR “resilience interventions”[All Fields] OR “resilience-building program”[All Fields] OR “resilience-based intervention”[All Fields] OR resilience-train*[All Fields] OR “psychological intervention"[All Fields] OR "psychological interventions"[All Fields] OR “building”[All fields] OR “enhance*”[All fields] OR “train*”[All fields] OR “educat*”[All fields] OR “teach*”[All fields] OR “increas*”[All fields] OR “promot*”[All fields] OR “prevent*”[All fields]) AND ("Internet based"[All fields] OR "Online"[All fields] OR "Online Therapy"[All fields] OR "Internet"[All fields] OR "Internet"[MeSH Terms] OR "Internet treatment"[All fields] OR "Web based"[All fields] OR "Internet intervention"[All fields] OR "Internet delivered"[All fields] OR "iCBT"[All fields] OR "website"[All fields] OR "computer"[All fields] OR "computer based"[All fields] OR "computer delivered"[All fields] OR "computer administered"[All fields] OR "technology"[All fields] OR "technology assisted"[All fields] OR "ICT"[All fields] OR "computerized"[All fields] OR "computerised"[All fields] OR "TELEhealth"[All fields] OR "eHealth"[All fields] OR "mobile"[All fields] OR "smartphone"[All fields] OR "tablet"[All fields])

**Google Scholar:**

(“Resilience” OR “hardiness” OR “cope” OR “coping” OR “psychological resilience”) (“psychological intervention” OR "psychological interventions" OR “building” OR “enhance” OR “train” OR “education” OR “teach” OR “increase” OR “promote” OR “prevent” OR “intervention” OR “interventions”) ("Internet based" OR "Online" OR "Online Therapy" OR "Internet" OR "Internet" OR "Internet treatment" OR "Web based" OR "Internet intervention" OR "Internet delivered" OR "iCBT" OR "website" OR "computer" OR "computer based" OR "computer delivered" OR "computer administered" OR "technology" OR "technology assisted" OR "ICT" OR "computerized" OR "computerised" OR "TELEhealth" OR "eHealth" OR "mobile" OR "smartphone" OR "tablet")
