# Supplementary material for: Theoretical adequacy, methodological quality and efficacy of online interventions targeting resilience: a systematic review and meta-analysis
Source: Eur J Public Health. 2021 Jul 7;31(Suppl 1):i11–8. doi: 10.1093/eurpub/ckaa255 (PMC8266533; doi:10.1093/eurpub/ckaa255)
Supplement: ckaa255_Supplementary_Data [file ckaa255_supplementary_data.zip › ckaa255-suppl_data/S3_Summary of characteristics of studies included.docx]

**S3**. Summary of characteristics of RCTs included

| Author,  Year | Purpose | Participants, | Age (mean) | % of women in the total sample | Randomized | Design | Intervention Description | Definition of Resilience | Theoretical Basis | Outcomes |
| --- | --- | --- | --- | --- | --- | --- | --- | --- | --- | --- |
| Abbott et al.,  2009 | To enhance resilience by teaching seven skills to help improve ability to cope with challenges and setbacks and maximize potential achievements | Sales managers volunteers, | IG: 40.50 (9.45)  CG: 46.00 (9.99) | 13.2% (IG: 15.4%;  CG: 11.1%) | IG: 26  WL: 27 | PRE, POST, FU (10 weeks after POST; data not presented in the paper) | Name: ROL  Format: Internet-based program (video, slides, Virtual Partners, graphical feedback, emails and a conference call)  Duration: 10 week content/Training components:  - Emotion regulation  - Impulse control  - Optimism  - Causal analysis  - Empathy  - Self-efficacy  - Reaching out | A person’s ability to persevere in the face of challenges, setbacks and conflicts (Reivich & Shatte, 2002) | Cognitive therapy | Happiness:  - AHI  Quality of life:  - WHOQOL-BREF  Depressive, anxiety, and stress symptoms:  - DASS-21  Statistics for work performance  Satisfaction:  - ROL Satisfaction Questionnaire |
| Acosta et al.,  2017 | To developed and evaluated a web-based self-management intervention based in CBT, targeting PTSD symptoms and hazardous substance use | Veterans with PTSD/ subthreshold PTSD and hazardous substance use, | 34.00 (8.1,) (range=22-64); | 7% | IG: 81  TAU: 81 | PRE, POST, FU (1-and 3 month after POST) | Name: Thinking Forward.  Format: Web-based program (Interactive exercises, graphs , a printable workbook and fillable copies of exercises)  Duration: 12 weeks  Content/Training components: 24 modules (12 core, 12 additional; approximately 20 minutes each):  - Cognitive behavioral skills  - Relaxation  - Emotional centering | Resilience defined as having the coping resources to thrive in the face of adversity | CBT | Eligibility and baseline health status:  - CAPS  - AUDIT  - DAST-10  - MINI  Primary study outcomes:  - TLFB  - PCL-M  - WHOQOL-BREF  Additional outcomes:  - CSS  - MOS-SSS  - BSCQ  - Future Scale  - RTCQ  - CD-RISC |
| Aikens et al.,  2014 | Examine mindfulness program to decrease employee stress and enhance resiliency and well-being | Population of general employees at The Dow Chemical Company (Dow), | Age range= 18-65 | NR | IG: 44  WL: 45 | PRE, POST, FU (6 months after POST) | Name: The mindfulness intervention  Format: online (via webinar, the Internet or cell phone; Audio exercises, pre-programmed e-mail coaching and feedback specific, and text messaging system)  Duration: 7 weeks  Content/Training components:  - Mindfulness | NR | MBSR | Mindfulness:  - FFMQ  Stress:  - PSS-14  Resilience:  - CD-RISC  Vigor and work engagement:  - Shirom Vigor Scale  Lifestyle behaviors:  - Lifestyle Survey Questions |
| Bekki et al.,  2013 | Online personal resilience training program for women in stem doctoral programs | Female doctoral students in the physical sciences and engineering, | 27.3 (range=22-52); | 100% | IG: 66  WL: 68 | PRE, POST (after 5 week training) | Name: CareerWISE website  Format: online  Duration: 2-weeks  Content/Training components: Instructional modules, informational briefs, video interviews, self-tests, and practice exercises.  - Self-efficacy  - Problem-solving  - Cognitive-behavioral skills. | Resilience is a multidimensional construct that has been applied to self-assessments of current, dispositional, or past emotions, beliefs, and behaviors and to stress levels and coping skills (Lightsey, 2006). Resilience is a dynamic process (American Psychological Association, http://www.apa.org/helpcenter/road-resilience.aspx) that has been studied by focusing on linked variables or patterns of individual adaptation (Masten, 2001). | Grounded in psychological theory and research on personal resilience and coping in the face of stressors | - Problem-Solving Knowledge Scale  - Resilience Scale,  - Coping Efficacy Scale  - Personal Resources Scale  - Confidence to Achieve STEM Landmarks Scale  - Coping Styles  - Barrier perceptions |
| De Voogd et al.,  2016 | Online attention bias modification training to reduce attention bias and symptoms of anxiety and depression and to increase emotional resilience in youth | Unselected adolescents, | 14.41 (1.20) | 57.6% ((VS: 58.7%, DP: 56.3%, VS Placebo: 63.2%, DP Placebo: 54.2%) | VS = 126, DP = 128, VS Placebo = 38, DP Placebo = 48 | PRE, POST, FU (at 3, 6, and 12 months after POST) | Name: CBM-A  Format: online  Duration: 4 weeks  Content/Training: 8 sessions (VS: 15 minutes; DP: 8 minutes)  - Visual search attention training  - Dot-probe attention training | NR | Based on diathesis-stress theory (Beck, 1967) | Primary outcome measures:  - SCARED  - CDI  Secondary outcome measures:  - RSES  - PTQ  - PMT-K  - SDQ  - ACS |
| De Voogd et al., 2017a | To test a training aimed to modify interpretation bias to promote emotional resilience | Adolescents with heightened symptoms of anxiety and/ or depression, | 14.45 (1.53); | 66.7% (VS: 63.2%,  VS Placebo: 65.6%,  No-training control group: 71.1%) | VS: 38,  VS Placebo: 32,  No-training control group: 38 | PRE, POST, FU (at 3 and 6 months after POST) | Name: Visual search attentional bias modification  Format: online  Duration: 4 weeks  Content/Training: 8 sessions  - Visual Search training | NR | Visual Search Task | Primary outcome measures:  - SCARED  - CDI  Secondary outcome measures:  - RSES  - PTQ  - SDQ-P |
| De Voogd et al.,  2017b | To test a training aimed to modify interpretation bias to promote emotional resilience | Adolescents with heightened symptoms of anxiety or depression, | 15.68 (1.33); | 63% (Scenario: 63.9%, Picture-word: 59.1%, Neutral control training: 66.7%) | Scenario: 36, Picture-word: 44, Neutral Control training: 39 | PRE, POST, FU (at 3 and 6 months after POST) | Name: CBM-I  Format: online  Duration: 4 weeks  Content/Training: 8 sessions (approximately 15 minutes each)  - Text-based scenario training  - Picture- word imagery training | NR | NR | Primary outcome measures:  - SCARED  - CDI  Secondary outcome measures:  - REC-T  - SST  - RSES  - PTQ  - SDQ-P  - SUIS |
| Hoorelbeke et al.,  2015 | To examine whether working memory based CCT can heighten resilience to stress and reduce rumination in the wake of stress | At-risk undergraduate students (high trait rumination), | IG: 20.84 (2.27);  Active control: 20.45 (1.45) | 62% (  IG: 100%;  Active control: 18.1%) | IG: 25  Active control: 22 | PRE, POST, FU (4 weeks after POST) | Name: Cognitive control training  Format: online  Duration: 14 days  Content/Training: 10 training sessions.  - Cognitive control training  - Visual search training | Resilience, as operationalized by stress reactivity and rumination in response to a lab stressor | Working memory functioning as a way to reduce cognitive vulnerability for depression | Depressive symptomatology:  - BDI-II-NL  Depressive and anxious symptomatology:  - MASQ-D30  Rumination:  - RRS-NL-EXT  Worrying:  - PSWQ  Positive and negative affective states:  - PANAS  Attentional control:  - ACS-NL  Resilience:  - RS-NL |
| Hoorelbeke & Koster,  2016 | To test whether internet-delivered CCT can be used as an intervention to increase resilience to depression in RMD patients | Remitted depressed sample, | IG: 46.12 (10.80);  Active control: 47.82 (12.20) | 66.2% ( IG: 64,7%; Active control: 67,6% ) | IG: 34  Active control: 34 | PRE, POST, FU (3 months after POST) | Name: Cognitive control training  Format: online  Duration: 2 weeks  Content/Training: 10 sessions of the adaptive PASAT | NR | Working memory functioning as a way to reduce cognitive vulnerability for depression | Cognitive transfer:  - PASAT  - BRIEF-A  Primary outcome measures:  - RRS  - BDI-II  Secondary outcome measures:  - CERQ  - WHODAS 2.0  - QLDS  - RS  - RDQ  Other measures:  - LTE  - CEQ |
| Pauls et al.,  2016 | To examine whether mindfulness fosters resilience and reduces emotional exhaustion; evaluation of web-based mindfulness intervention | Employees at several German companies, | 41,00 (11,63); | 64% | IG: 57  Active control: 56 | PRE, POST | Name: Mindfulness intervention  Format: Web-based intervention  Duration: 4 days  Content/Training: 5 sessions (10 minutes each)  - Mindfulness | Resilience refers to personal resources and behavioral strategies that protect psychological well-being even under high demands | Classical mindfulness training | Mindfulness:  - FFMQ  - Short version of resilience questionnaire by Soucekt et al. (2015)  - Burnout Inventory |
| Rose et al.,  2013 | To enhance resilience and reduce stress among healthy but stressed graduate students | Graduate students with no psychiatric disorder and heightened stress (PSS > 16), | 27.32 (3.53) | 50% | IG: 34  Active control: 32 | PRE, POST | Name: SMART-OP (Stress Management and Resilience Training for Optimal Performance).  Format: Self-guided, online (interactive didactic video presentations, video feedback, stress briefing, feelings, thoughts and action activities, homework)  Duration: 6 weeks  Content/Training: 6 sessions  - Feeling activities  - Thoughts activities  - Action activities | “The ability of individuals to adapt successfully in the face of acute stress, trauma, or chronic adversity, maintaining or rapidly regaining psychological well-being and physiological homeostasis” | NR | Pre- and post-assessment self-report measures:  - PSS-10  - SPOCS  Other self-report measures:  - Stress Management Training Surveys  - SUS  - TSST  - Psychophysiological measures |

Note: ACS: Attentional Control Scale; AHI: Authentic Happiness Inventory; AUDIT: Alcohol Use Disorders Identification Test; BDI-II: Beck Depression Inventory; BRIEF-A: Behavior Rating Inventory of Executive Function Adult Version; BSCQ: Brief Situational Confidence Questionnaire; CAPS: Clinical Administered PTSD Scale; CBM-A: Cognitive Bias Modification of Attention; CBM-I: Cognitive bias modification of interpretations; CBT: Cognitive Behavioral Therapy; CCT: Cognitive Control Training; CDI: Children's Depression Inventory; CD-RISC: Connor-Davidson Resilience Scale; CEQ: Credibility/Expectancy Questionnaire; CERQ: Cognitive Emotion Regulation Questionnaire; CG: Control Group; CSS: Coping Strategies Scale; DASS-21: Depression Anxiety and Stress Scales; DAST-10: Drug Abuse Screening Test-10; DP: Dot-Probe attention training; FFMQ: Five Facets of Mindfulness Questionnaire; FU: Follow up (a period after the end of the program, waiting list or treatment as usual); IG: Intervention Group; LTE: List of Threatening Experiences; MASQ-D30: Mood and Anxiety Symptom Questionnaire; MBSR: Mindfulness Based Stress Reduction; MINI: MINI International Neuropsychiatric Interview; MOS-SSS: Medical Outcomes Study Support Scale; NR: Not Reported; PANAS: Positive and Negative Affect Schedule; PASAT: Paced Auditory Serial Addition Task; PCL-M: PTSD Checklist-Military; PMT-K: Performance Motivation Test for children; POST: Post-intervention (after the end of the program, waiting list or treatment as usual); PRE: Pre-intervention (prior to starting the program, waiting list or treatment as usual); PSS: Perceived Stress Scale; PSS-10: Perceived Stress Scale-10; PSS-14: Perceived Stress Scale-14; PSWQ: Penn State Worry Questionnaire; PTQ: Perseverative Thinking Questionnaire; PTSD: Post-Traumatic Stress Disorder; QLDS: Quality of Life in Depression Scale; RDQ: Remission of Depression Questionnaire; REC-T: Recognition Task; RMD: Remitted Depressed; ROL: ResilienceOnline; RRS: Ruminative Response Scale; RSES: Rosenberg Self-Esteem Scale; RTCQ: Readiness to Change Questionnaire; SCARED: Screen for Child Anxiety Related Emotional Disorders; SDQ: Strengths and Difficulties Questionnaire; SDQ-P: Strengths and Difficulties Questionnaire parent version; SPOCS: Stress and Perception of Control Scale; SST: Scrambled Sentence Task; SUIS: Spontaneous Use of Imagery Scale; SUS: System Usability Scale; TAU: Treatment As Usual; TLFB: Timeline Follow-Back; TSST: Trier Social Stress Test; VS: Visual Search attention training; WHODAS: World Health Organization Disability Assessment Schedule 2.0; WHOQOL-BREF: World Health Organization Quality of Life – BREF; WL: Waiting List
