# Supplementary figures and images for: Theoretical adequacy, methodological quality and efficacy of online interventions targeting resilience: a systematic review and meta-analysis
Source: Eur J Public Health. 2021 Jul 7;31(Suppl 1):i11–8. doi: 10.1093/eurpub/ckaa255 (PMC8266533; doi:10.1093/eurpub/ckaa255)

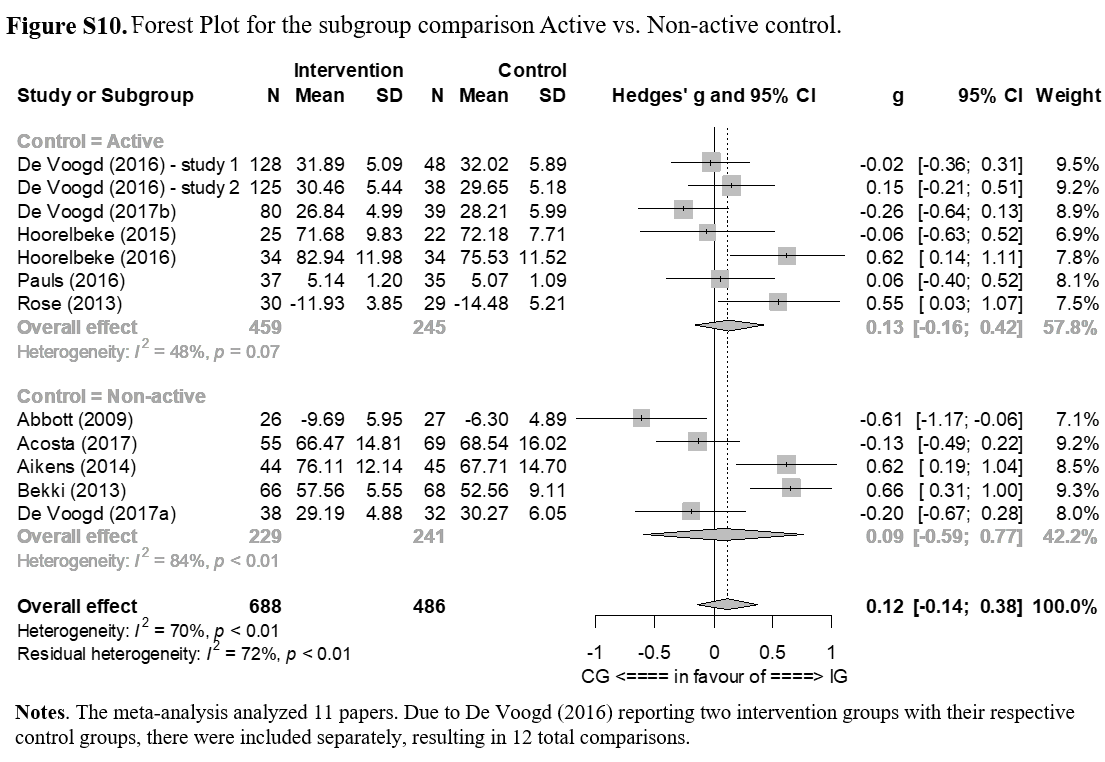

Supplement: ckaa255_Supplementary_Data [file ckaa255_supplementary_data.zip › ckaa255-suppl_data/S10_Subgroup_Control.tif]

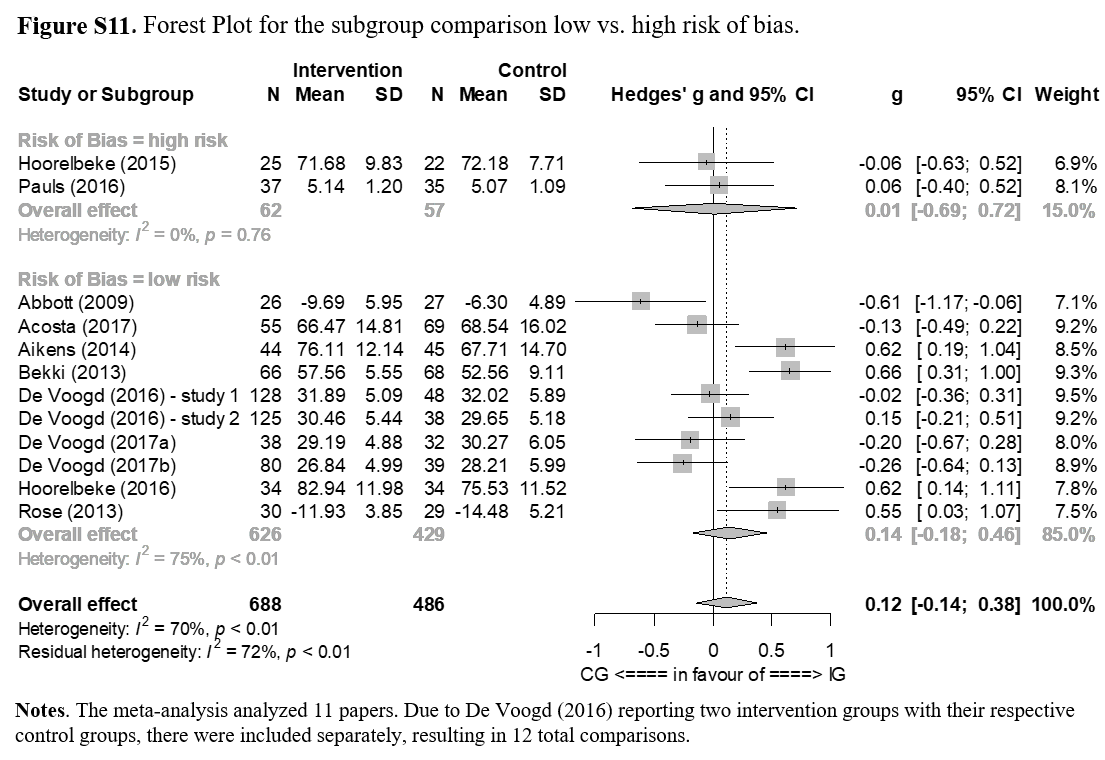

Supplement: ckaa255_Supplementary_Data [file ckaa255_supplementary_data.zip › ckaa255-suppl_data/S11_Subgroup_RoBGroup.tif]

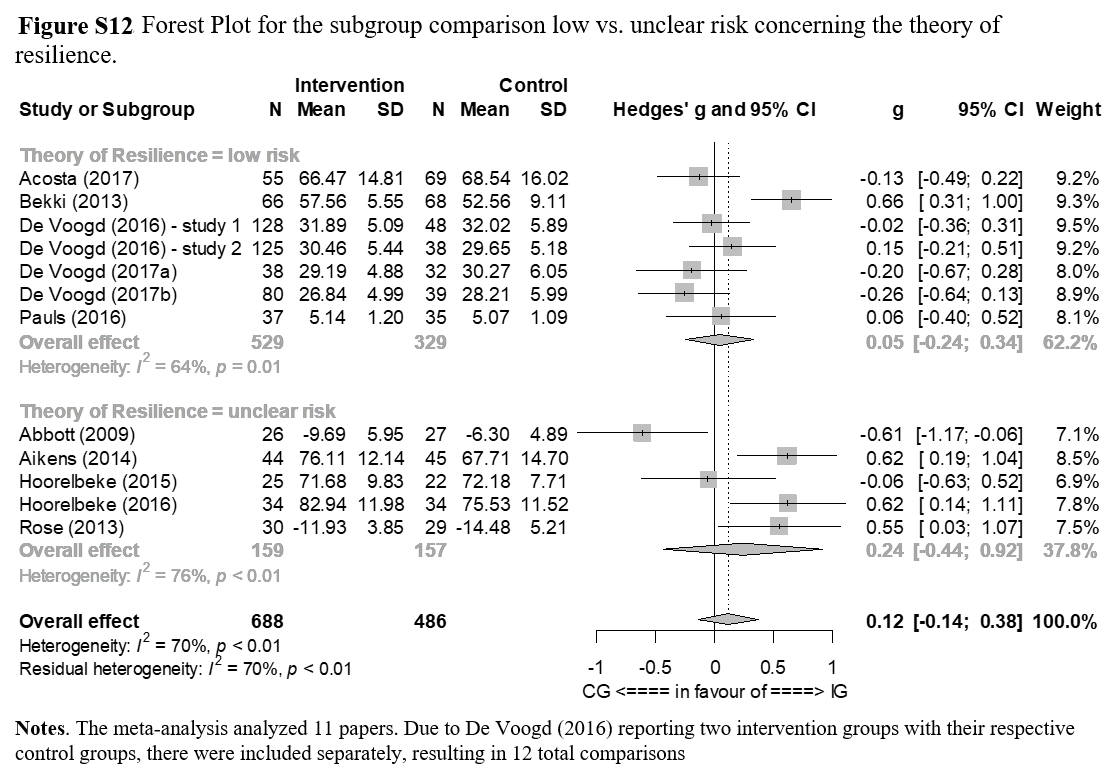

Supplement: ckaa255_Supplementary_Data [file ckaa255_supplementary_data.zip › ckaa255-suppl_data/S12_Subgroup_TheoryResilience.tif]

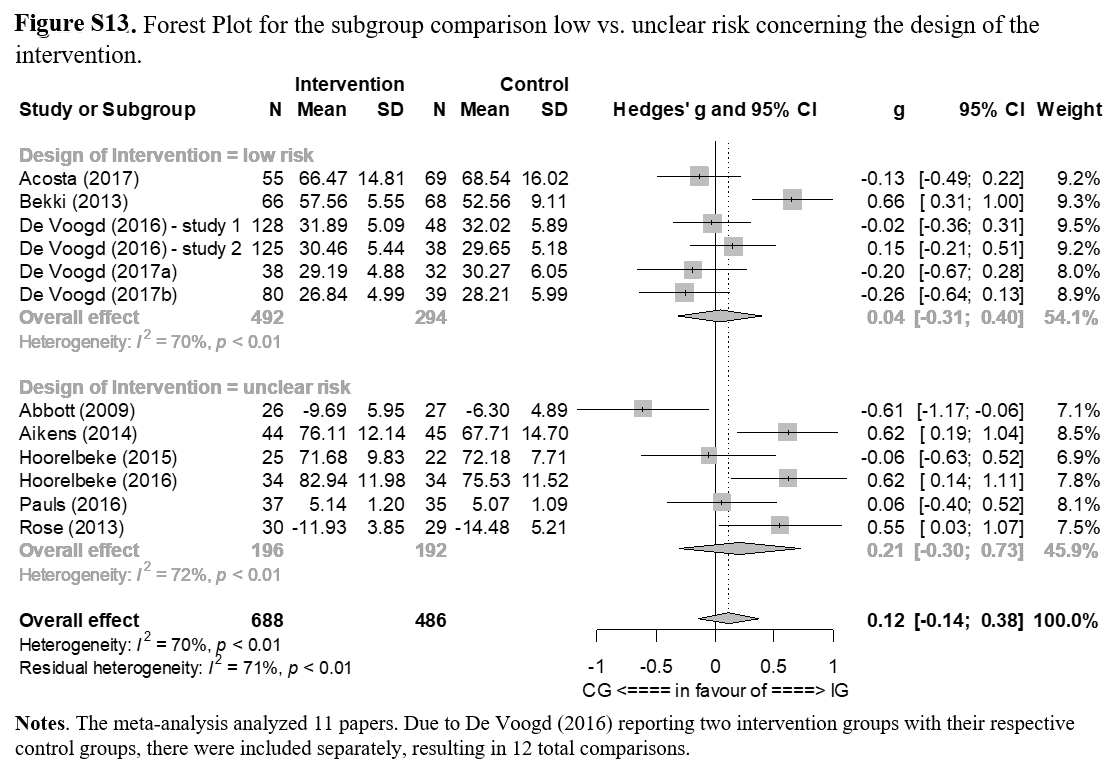

Supplement: ckaa255_Supplementary_Data [file ckaa255_supplementary_data.zip › ckaa255-suppl_data/S13_Subgroup_DesignOfIntervention.tif]

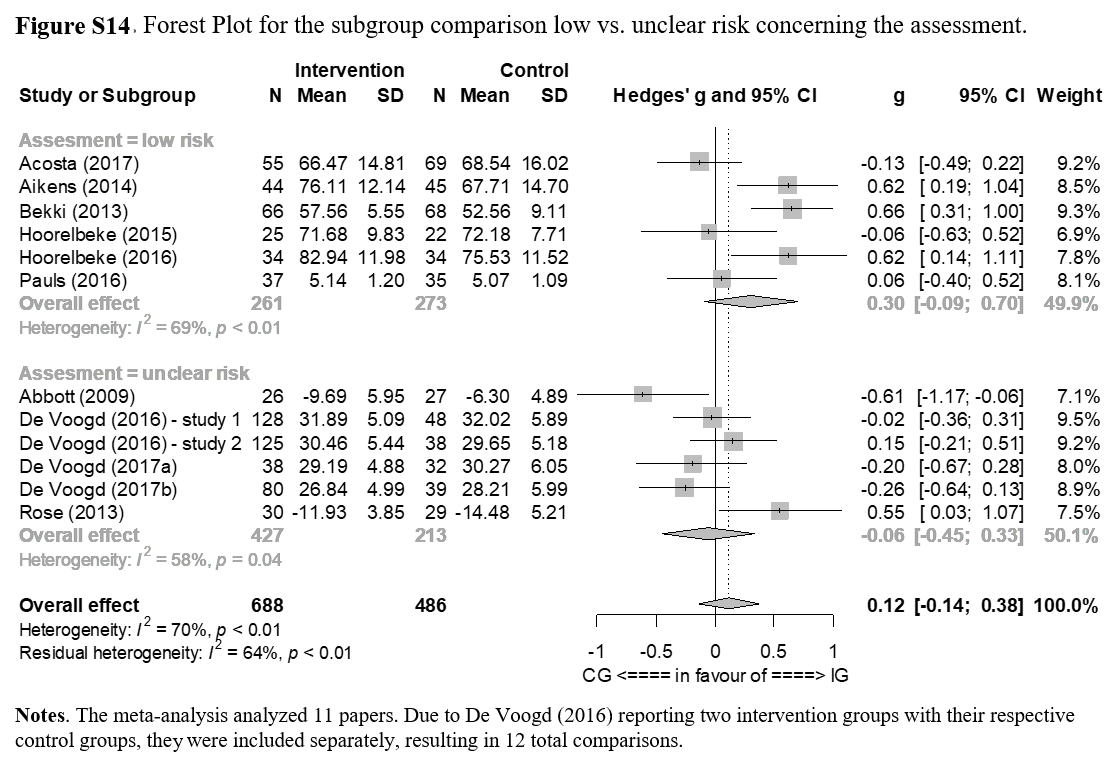

Supplement: ckaa255_Supplementary_Data [file ckaa255_supplementary_data.zip › ckaa255-suppl_data/S14_Subgroup_Assesment.tif]

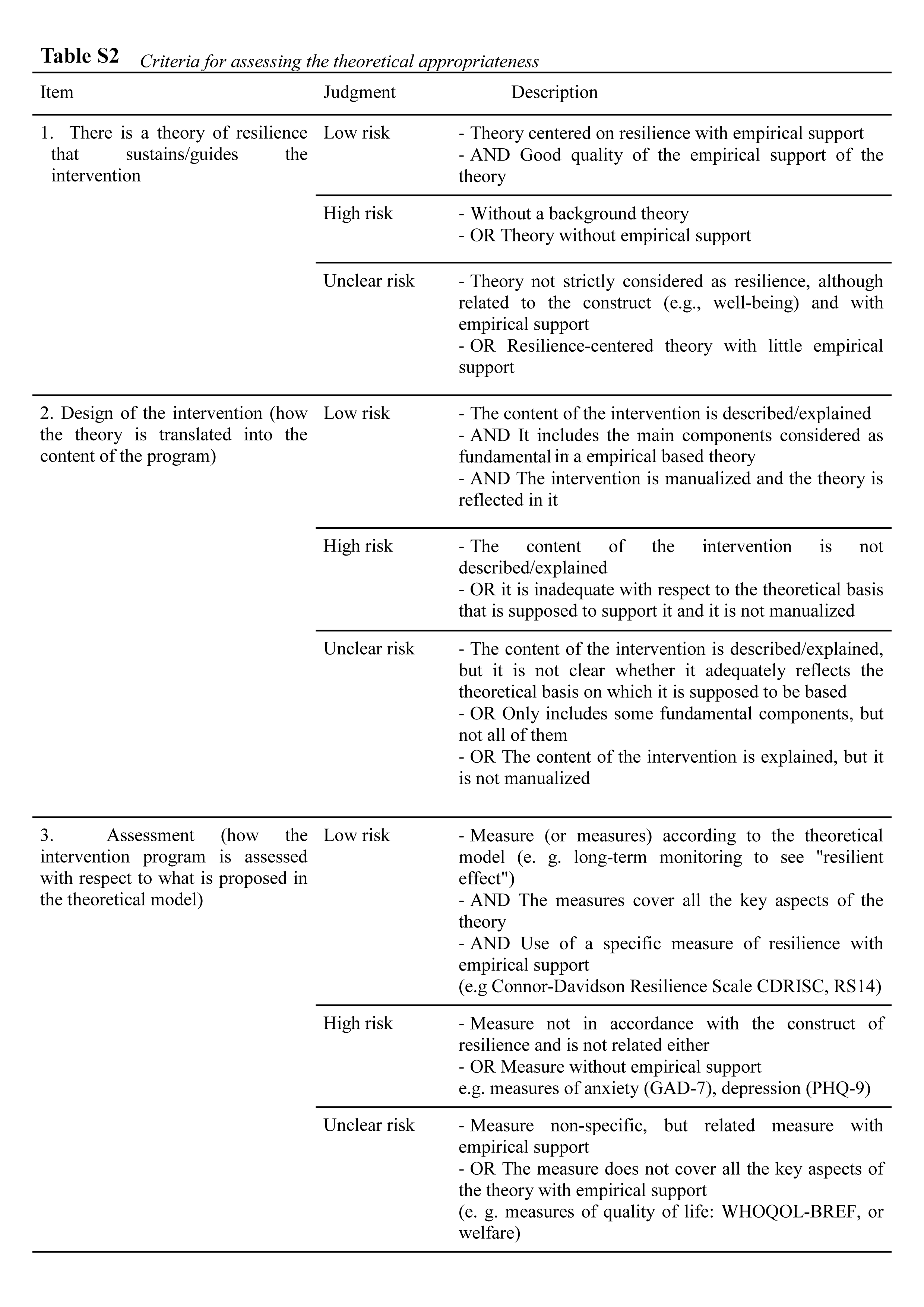

Supplement: ckaa255_Supplementary_Data [file ckaa255_supplementary_data.zip › ckaa255-suppl_data/S2_Criteria for assessing the theoretical appropriateness (1).tif]

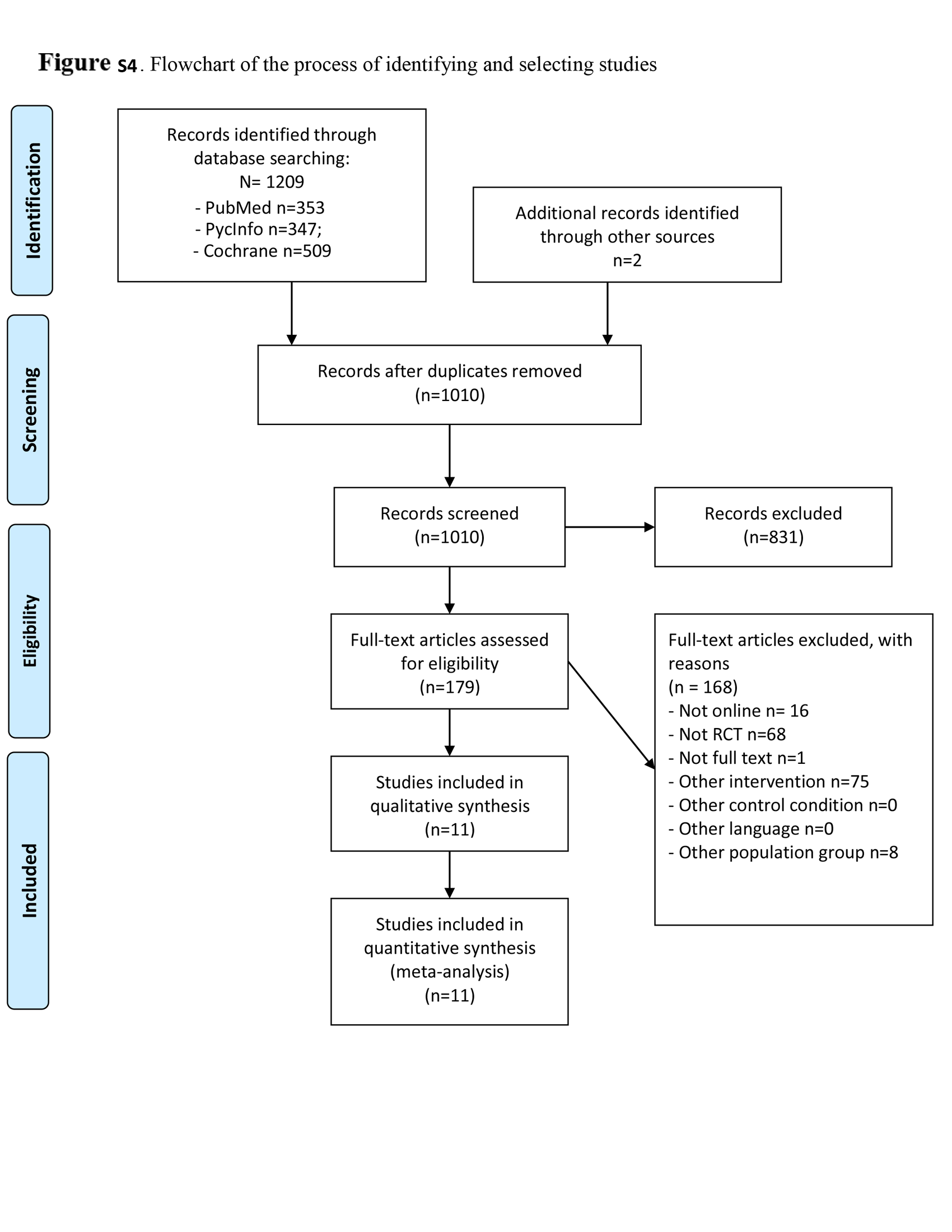

Supplement: ckaa255_Supplementary_Data [file ckaa255_supplementary_data.zip › ckaa255-suppl_data/S4_Flowchart.tiff]

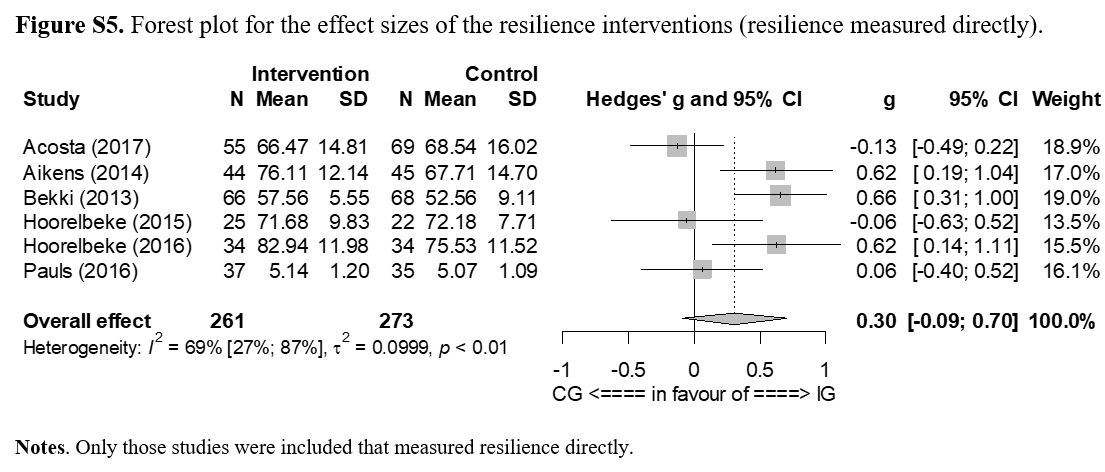

Supplement: ckaa255_Supplementary_Data [file ckaa255_supplementary_data.zip › ckaa255-suppl_data/S5_SensitivityAnalysis_Direct.tif]

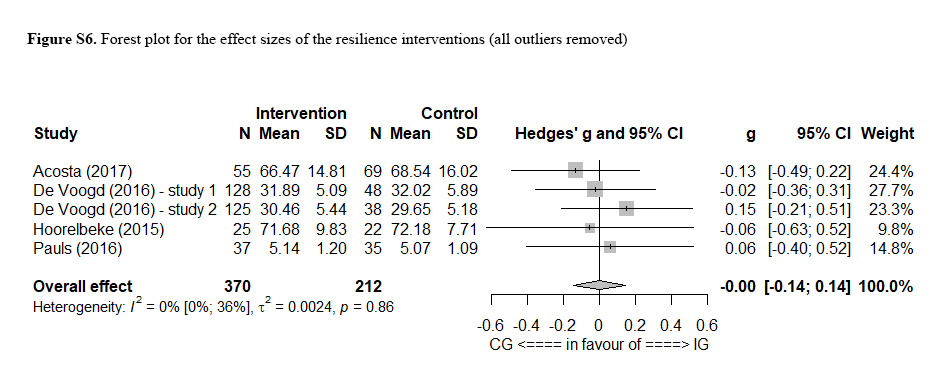

Supplement: ckaa255_Supplementary_Data [file ckaa255_supplementary_data.zip › ckaa255-suppl_data/S6_ Forest plot for the effect sizes of the resilience interventions (all outliers removed).tiff]

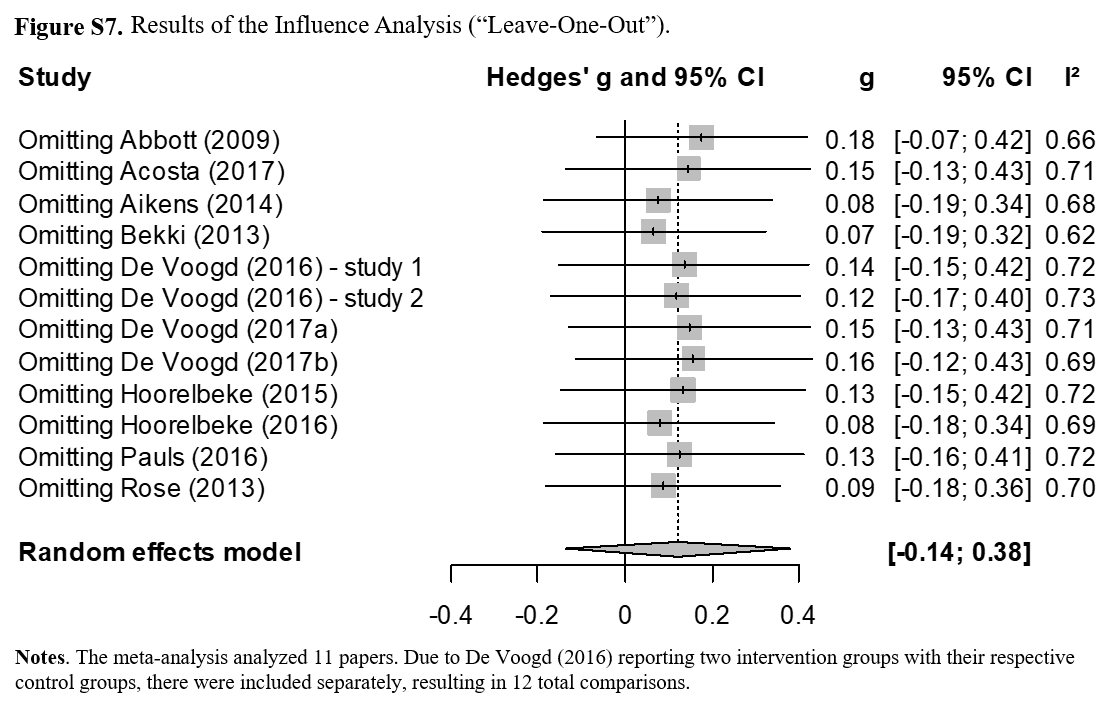

Supplement: ckaa255_Supplementary_Data [file ckaa255_supplementary_data.zip › ckaa255-suppl_data/S7_LeaveOneOut.tif]

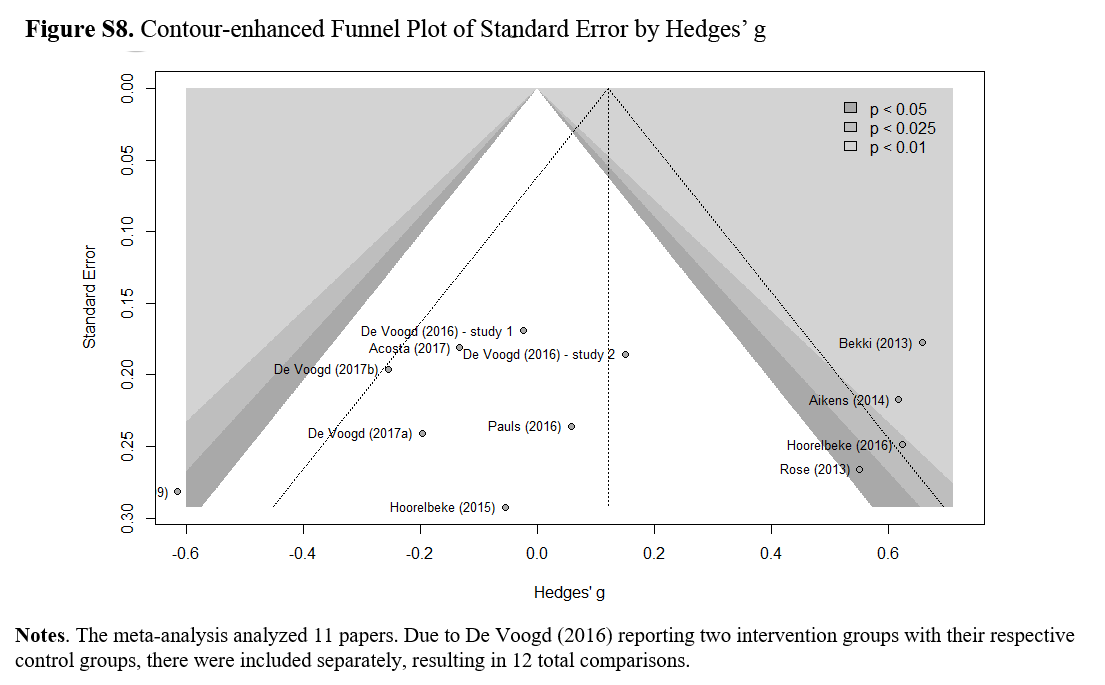

Supplement: ckaa255_Supplementary_Data [file ckaa255_supplementary_data.zip › ckaa255-suppl_data/S8_ Contour-enhanced Funnel Plot of Standard Error by Hedges g.tif]

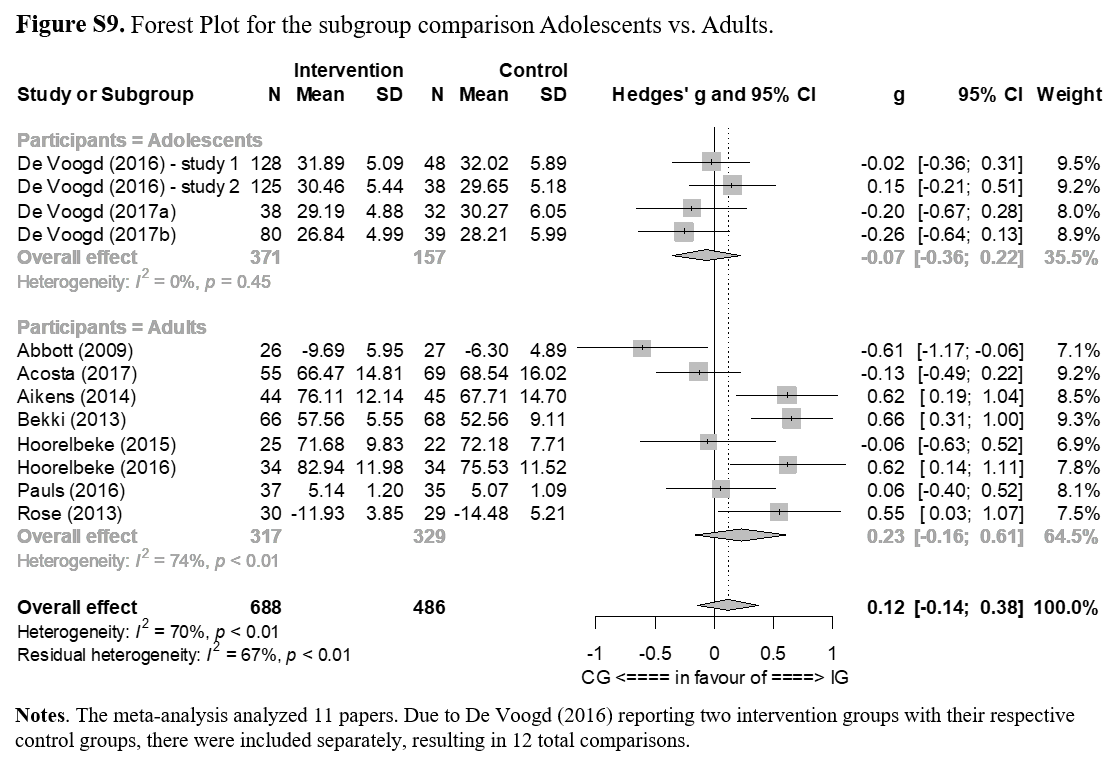

Supplement: ckaa255_Supplementary_Data [file ckaa255_supplementary_data.zip › ckaa255-suppl_data/S9_Subgroup_Participants.tif]
